# Genome-wide association study and genetic mapping of *BhWAX* conferring mature fruit cuticular wax in wax gourd

**DOI:** 10.1186/s12870-022-03931-z

**Published:** 2022-11-19

**Authors:** Jinqiang Yan, Feng Chen, Piaoyun Sun, Wenrui Liu, Dasen Xie, Yulei Qian, Biao Jiang

**Affiliations:** 1 Vegetable Research Institute, Guangdong Academy of Agricultural Sciences, Guangzhou, 510640 Guangdong China; 2 Guangdong Key Laboratory for New Technology Research of Vegetables, Guangzhou, 510640 Guangdong China

**Keywords:** Wax gourd, Fruit, Cuticular wax, GWAS, Genetic mapping, MBOAT

## Abstract

**Background:**

Wax gourd [*Benincasa hispida* (Thunb) Cogn. (2*n* = 2*x* = 24)] is an economically important vegetable crop of genus *Benincasa* in the Cucurbitaceae family. Fruit is the main consumption organ of wax gourd. The mature fruit cuticular wax (MFCW) is an important trait in breeding programs, which is also of evolutionary significance in wax gourd. However, the genetic architecture of this valuable trait remains unrevealed.

**Results:**

In this study, genetic analysis revealed that the inheritance of MFCW was controlled by a single gene, with MFCW dominant over non-MFCW, and the gene was primarily named as *BhWAX*. Genome-wide association study (GWAS) highlighted a 1.1 Mb interval on chromosome 9 associated with MFCW in wax gourd germplasm resources. Traditional fine genetic mapping delimited *BhWAX* to a 0.5 Mb region containing 12 genes. Based on the gene annotation, expression analysis and co-segregation analysis, *Bhi09G001428* that encodes a membrane bound O-acyltransferase (MBOAT) was proposed as the candidate gene for *BhWAX.* Moreover, it was demonstrated that the efficiency of a cleaved amplified polymorphic sequences (CAPS) marker in the determination of MFCW in wax gourd reached 80%.

**Conclusions:**

In closing, the study identified the candidate gene controlling MFCW and provided an efficient molecular marker for the trait in wax gourd for the first time, which will not only be beneficial for functional validation of the gene and marker-assisted breeding of wax gourd, but also lay a foundation for analysis of its evolutionary meaning among cucurbits.

**Supplementary Information:**

The online version contains supplementary material available at 10.1186/s12870-022-03931-z.

## Background

In nature, plant surface is covered by cuticles, which consist of non-polymerized cuticular waxes and cutins synthesized in epidermal cells [1]. Cutins are made up of ester bonds linked C16 and C18 fatty acid and their derivatives, as well as some glycerol and phenolic acids [2]. Cuticular waxes are mainly composed of very long chain fatty acids (VLCFAs) with C20-C34 chains and their derivatives, such as alcohols, esters, aldehydes, alkanes and ketones [3]. The composition of cuticular waxes differs between plant species as well as between organs in the same plant.

The synthesis of wax components is relatively not complex and has been extensively studied in model plant *Arabidopsis*. In plastids, C16- and C18-fatty acids that are synthesized de novo act as precursors, which are hydrolyzed by fatty acyl-acyl carrier protein thioesterase and then transported into the cytoplasm [4]. Then, they are catalyzed into C16 or C18 fatty acyl-CoAs by fatty acid synthase and exported to the endoplasmic reticulum [5]. Later, the C16 or C18 fatty acyl-CoAs are further elongated to very long chain (C24-C36) acyl-CoAs by fatty acid elongase (FAE) [3, 6], followed by conversion to primary alcohols and wax esters via alkane pathway and to alkanes and their derivatives via primary alcohol pathway, respectively [3, 6]. Many genes have been identified to be involved in cuticular wax synthesis. A couple of FAE genes including *KCS1*, *KCS2*, *KCS9*, *KCS16, KCS20* and *KCR1* that play roles in VLCFA elongation are essential for wax biosynthesis [7–11]. Other genes such as *CER1, CER1-LIKE1, CER2-like, CER3* and *CER26* that modify the structure of VLCFAs are also vital for cuticular wax synthesis [12–16].

In addition, the synthesis of cuticular waxes can also be regulated at the transcriptional, posttranscriptional and posttranslational levels [4]. According to reports, WIN1/SHN1, SHN2, SHN3, CFL1, HDG1 and several MYB transcription factors directly or indirectly affect the expression of genes involved in the cuticular wax biosynthesis at the transcriptional level; *CER7*, *WAR3/RDR1* and *WAR4/SGS3* regulate cuticular wax synthesis by affecting mRNA stability at the posttranscription level. *CER9* influences cuticular wax composition probably by changing the structure of proteins produced during wax synthesis process at the posttranslational level.

The inheritance of cuticular wax on the leaf surface of many plant species has been extensively investigated. In some cases, the trait is controlled by quantitative trait loci [17, 18], and in most cases, the genetic architecture of cuticular waxes is dominantly controlled by a single gene [19–23]. Many dominant loci have been successfully mapped using a forward mapping method, and *CER2* [23] and *GDSL-like lipase/acylhydrolase* [22] are considered as candidate genes for wax synthesis. In cucurbits, cuticular waxes exist on the fruit surface of many species and is a prominent appearance quality trait valued in the breeding program. The density of fruit waxes differs in cucumber cultivars. According to the genotype generated by polymorphic SSR markers, the cucumber cultivars without fruit waxes can be distinguished from those with fruit waxes based on the principle analysis [24]. *CER1* and *WAX2* in cucumber are induced by low temperature, drought, salt stress and ABA, and these two genes influence the biosynthesis of very long chain alkanes, a predominant wax component in cucumber [25, 26]. Moreover, the grafting with pumpkin onto cucumber also affects the biosynthesis of fruit wax esters in cucumber, and this might be regulated by an AP2/ERF-type transcription factor *CsWIN1* [27]. Hydrocarbons are the most abundant chemicals, followed by alcohols in the wax of watermelon fruit surface [28]. The waxy phenotype of watermelon is controlled by a single dominant gene, located in a 1.7 Mb physical interval based on BSA-seq, and genes including *ECR* are probable candidates for the trait [28].

Wax gourd [*Benincasa hispida* (Thunb) Cogn. (2*n* = 2*x* = 24)], the only member of genus *Benincasa* in the Cucurbitaceae family, is an economically important vegetable crop mainly cultivated in China, India, Japan and many other tropical, subtropical and temperate countries. The same as most of cucurbits, fruit is the main consumption organ of wax gourd. In view of the physiological existence of mature fruit cuticular wax (MFCW), wax gourd can be divided into two sub-groups, Fenpi Donggua (wax gourd with MFCW) and Qingpi Donggua (wax gourd without MFCW), which has been confirmed by evolutionary analysis based on the re-sequencing of wax gourd germplasm resources [29]. Apart from the evolutionary significance, the MFCW is also an important trait in the breeding program of wax gourd because of individual preference of consumers. However, until now, the chemical composition as well as the genetic basis for this important trait remains unknown. In this study, a genome-wide association study (GWAS) was firstly performed to localize the major MFCW locus. Thereafter, two wax gourd inbred lines, one with thick MFCW and the other without MFCW, were used to generate populations for genetic analysis and genetic mapping of the gene conferring this trait. The study will not only highlight the genetic basis of MFCW of wax gourd and promote the breeding of wax gourd cultivars with appealing appearance, but also shed light on the evolution basis of cuticular wax on the fruit surface of cucurbits.

## Results

### *Phenotypic characterization of MFCW between P*_*1*_* and P*_*2*_

**Fig. 1:**
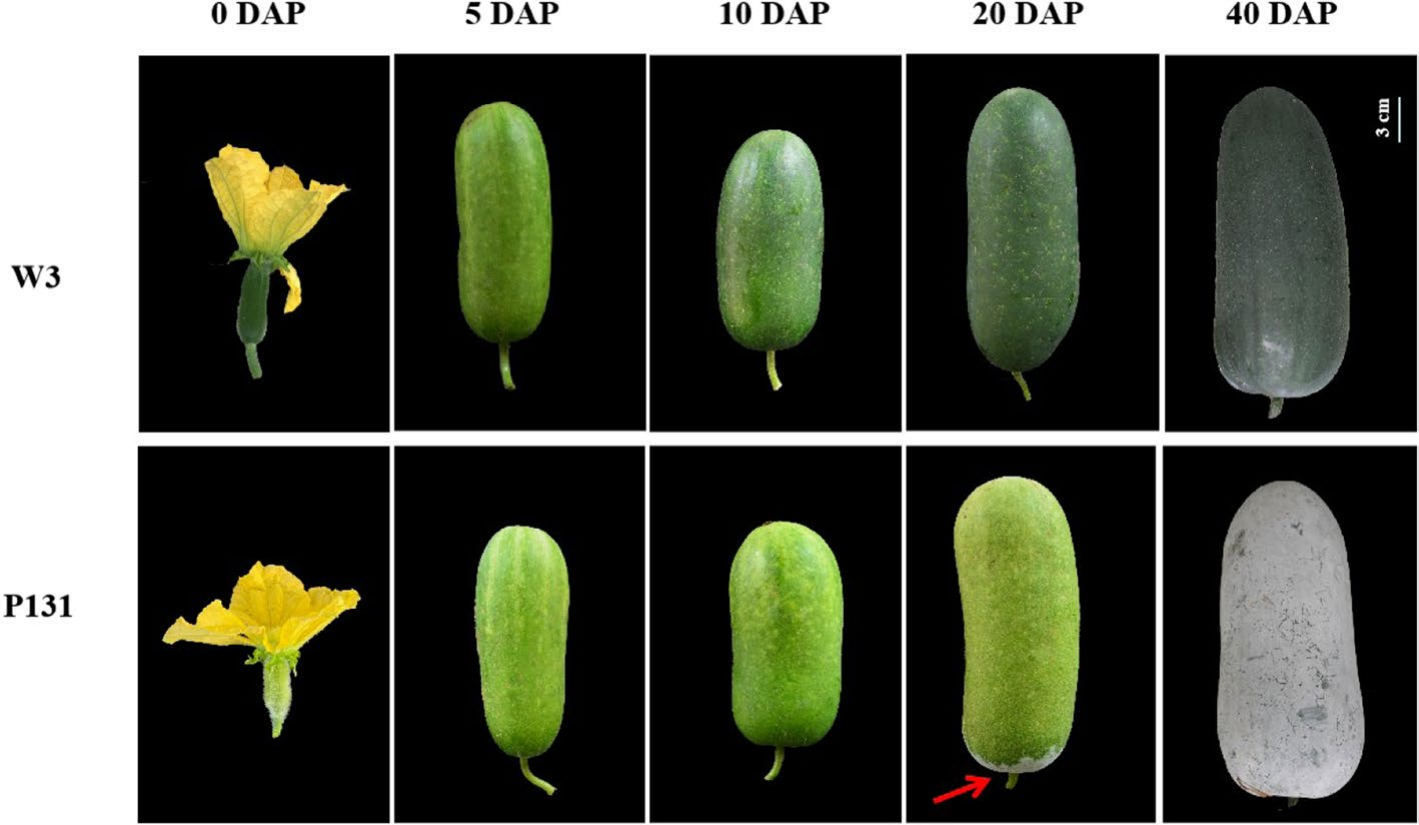
Fruit phenotype of P131 and W3 at different days after pollination (DAP). The red arrow indicated the cuticle wax around the fruit pedicel of P131 at 20 DAP

To further characterize the formation of cuticular waxes on the fruit surface of wax gourd, the fruit at different developmental stages of both P_1_ and P_2_ were phenotypically observed. It turned out that no MFCW was observed at any developmental stages of P_2_ and at 0, 5 and 10 DAP of P_1_. At 20 DAP, MFCW could be easily seen at the fruit pedicel, and the fruit was fully covered with thick MFCW at 40 DAP (Fig. 1).

### Genetic analysis results of MFCW in wax gourd

According to genetic analysis results, MFCWs were detected in 43 individuals derived from BC_1_P_1_ and 44 out of 85 individuals derived from BC_1_P_2_, which did not deviate from 1: 1 segregation ratio (χ2 = 0.053, *p* = 0.8179). Among 574 F_2_ individuals tested, 430 exhibited MFCWs while 144 exhibited no MFCWs, fitting with the Mendelian inheritance model controlled by a single dominant gene (χ^2^ = 0.01, *p* = 0.9748) (Table 1). Taken together, it could be concluded that the inheritance of the MFCW in wax gourd was controlled by a single dominant gene, which was named as *BhWAX*.

**Table 1:** Segregation of MFCW trait among six-generation family

Generation	No. individuals	No. MFCW	No. Non-MFCW	Segregation ratio(with: without)	χ2	p
P131 (P_1_)	30	30	0	-		
W3 (P_2_)	30	0	30	-		
F_1_	30	30	0	-		
BC_1_P_1_	43	43	0	-		
BC_1_P_2_	85	44	41	1.073:1	0.053	0.8179
F_2_	574	430	144	2.986:1	0.001	0.9748

### GWAS results of MFCW in wax gourd

**Fig. 2:**
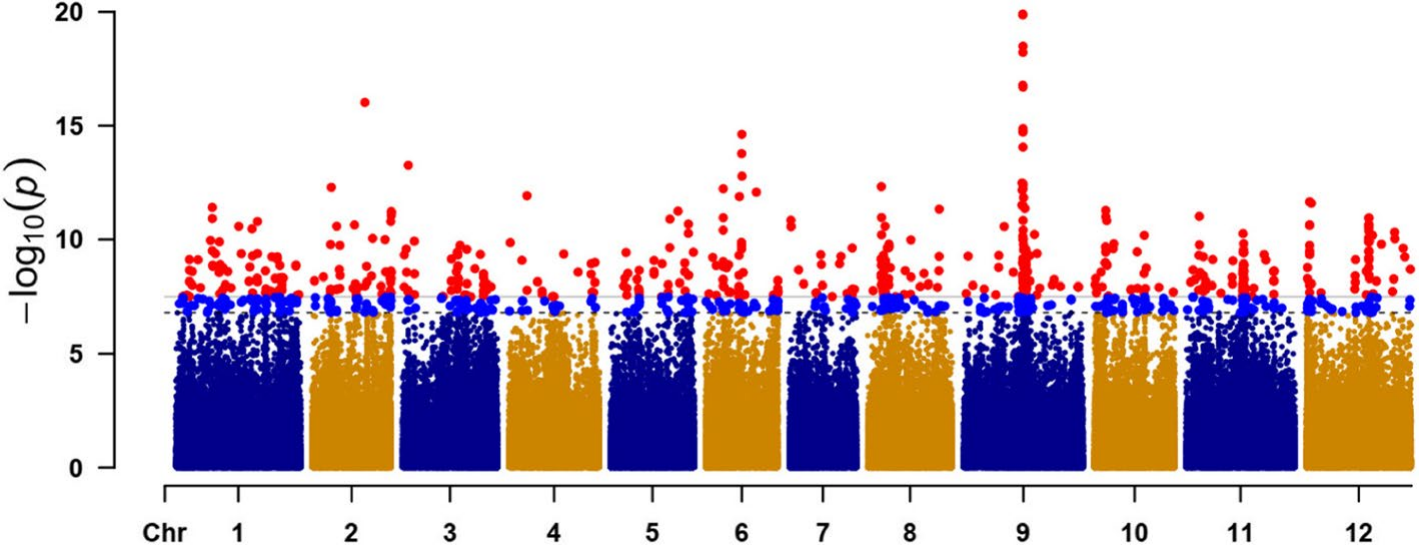
Manhattan plot of MFCW trait based on a genome-wide association study

According to the existence of MFCW and re-sequencing data of 146 wax gourd germplasm resources [29], GWAS was performed to decipher the genetic control of MFCW in wax gourd. The significant thresholds for SNP-based GWAS were set at 3.12022E-08 (0.01/320,490) (solid line) and 1.56011E-07 (0.05/320490) (dash line), respectively, where 320,490 SNPs were used for GWAS herein. A clear signal with an interval around 1.1 Mb (from 45,182,409 to 46,295,796 bp) on chromosome 9 was observed to show close correlation with MFCW based on the GWAS result (Fig. 2). Inside this interval, 24 genes (from *Bhi09M001405* to *Bhi09M001428*) were included according to Cucurbit Genomics Database (http://cucurbitgenomics.org/v2/organism/3).

### Genetic mapping results and candidate genes identified for BhWAX locus

**Fig. 3:**
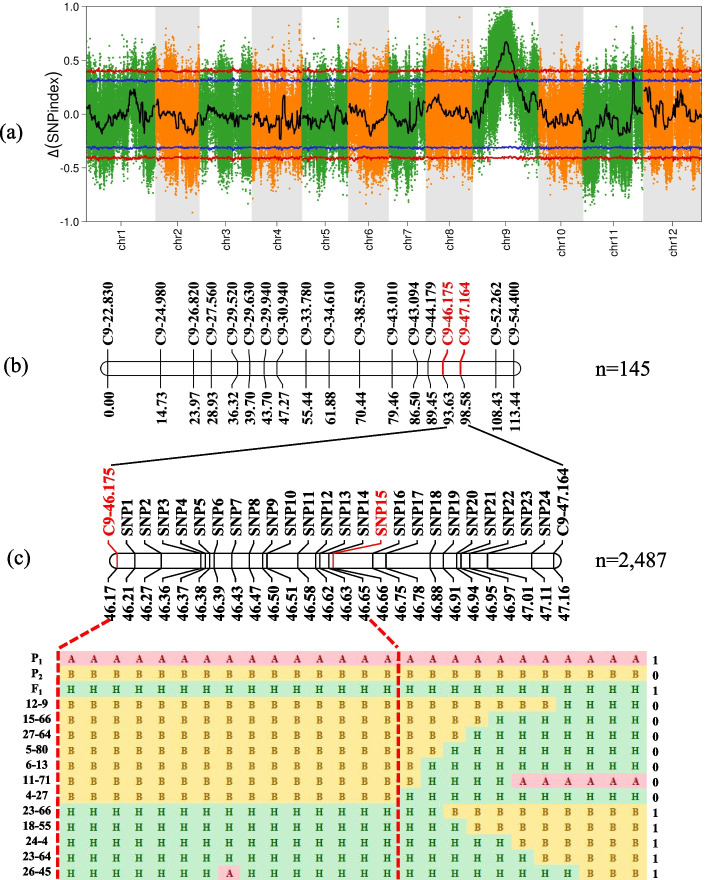
Genetic mapping of the MFCW gene *BhWAX* in wax gourd. **a** Δ(SNP index) graph generated from BSA-Seq. Blue lines and red lines indicated statistical significance surpass the threshold of 0.95 and 0.99 respectively. **b** Initial mapping of *BhWAX* gene using 145 F_2_ individuals. Names of markers were presented on the right side of the bar and genetic distance on the left side. **c** Fine mapping of *BhWAX* gene using 2,487 F_2_ individuals. Names of markers were presented on the right side of the bar and physical position of the marker on the left side. *BhWAX* gene was mapped between markers in red

To rapidly map the *BhWAX* locus, BSA-seq analysis was performed. Based on the phenotype of F_2_ individuals, they were randomly selected to construct F_2_-1 pool (30 individuals) and F_2_-0 pool (30 individuals), respectively. After the sequencing of the two pools, a total of 28.27, 30.38, 31.58 and 26.98 Gb raw data were generated for P_1_, P_2_, F_2_-1-pool and F_2_-0-pool, respectively. After raw data screening, the Q30 was all above 91% and the GC content was all around 37% (Table 2). In addition, a total of 1,451,083 SNPs were identified between two pools and used for BSA-seq analysis based on Δ(SNP-index) method (99% confidence interval). Furthermore, the results also revealed a strong signal peak on chromosome 9 covering about 27.92 Mb (31,580,001 bp—59,500,000 bp), displaying a correlation with *BhWAX* locus (Fig. 3).

**Table 2:** Sequencing statistics of BSA-seq samples

Sample	Raw reads	Clean reads	Q30 Percentage (%)	Mapped reads	Mapping rate (%)	Coverage 1X (%)	Coverage 4X (%)	Coverage 10X(%)
P131	187,937,372	171,855,518	91.49	171,713,436	91.37	95.35	94.22	89.83
W3	202,002,394	183,065,950	91.97	182,339,950	90.27	95.29	94.20	89.88
F_2_-1	209,992,928	192,113,997	92.33	192,362,254	91.60	95.78	94.90	91.14
F_2_-0	158,059,030	143,856,847	91.08	144,321,550	91.31	95.69	94.00	86.74

Based on the re-sequencing results of two parental lines, 159 Indel markers from linkage group 9 were developed. Among them, 56 markers were polymorphic and thereafter used for genotyping of 145 F_2_ individuals. After the removal markers with ambiguous amplification and distorted markers, the genotypes obtained by 18 informative markers were used for genetic mapping of *BhWAX* locus. Then the *BhWAX* locus was mapped between markers C9-46.175 and C9-47.164, with a genetic distance of 3.7 cM and 3.0 cM, respectively, corresponding to a physical interval ~ 0.99 Mb (46,175,172 bp-47,164,043 bp) (Fig. 3). The LOD value was 20.11, which could explain 42.03% of the phenotypic variance.

To further fine map the *BhWAX* locus, the same large F_2_ population comprising 2,487 individuals were genotyped using two flanking markers C9-46.175 and C9-47.164 in the spring of 2021. As a result, 46 recombinants were successfully identified and these recombinants were planted in the field until harvest for MFCW trait observation. Based on the genomic difference between two parental lines, 24 markers were further developed to genotype the 46 recombinants. As revealed by the genotyping and phenotyping results of 46 recombinants, the *BhWAX* locus was delimited to a 0.51 Mb interval (46,175,172 bp—46,685,864 bp) between marker C9-46.175 and maker SNP15 (The representative recombinant events see Fig. 3).

### Bhi09G001428 was the candidate gene for BhWAX locus

**Fig. 4:**
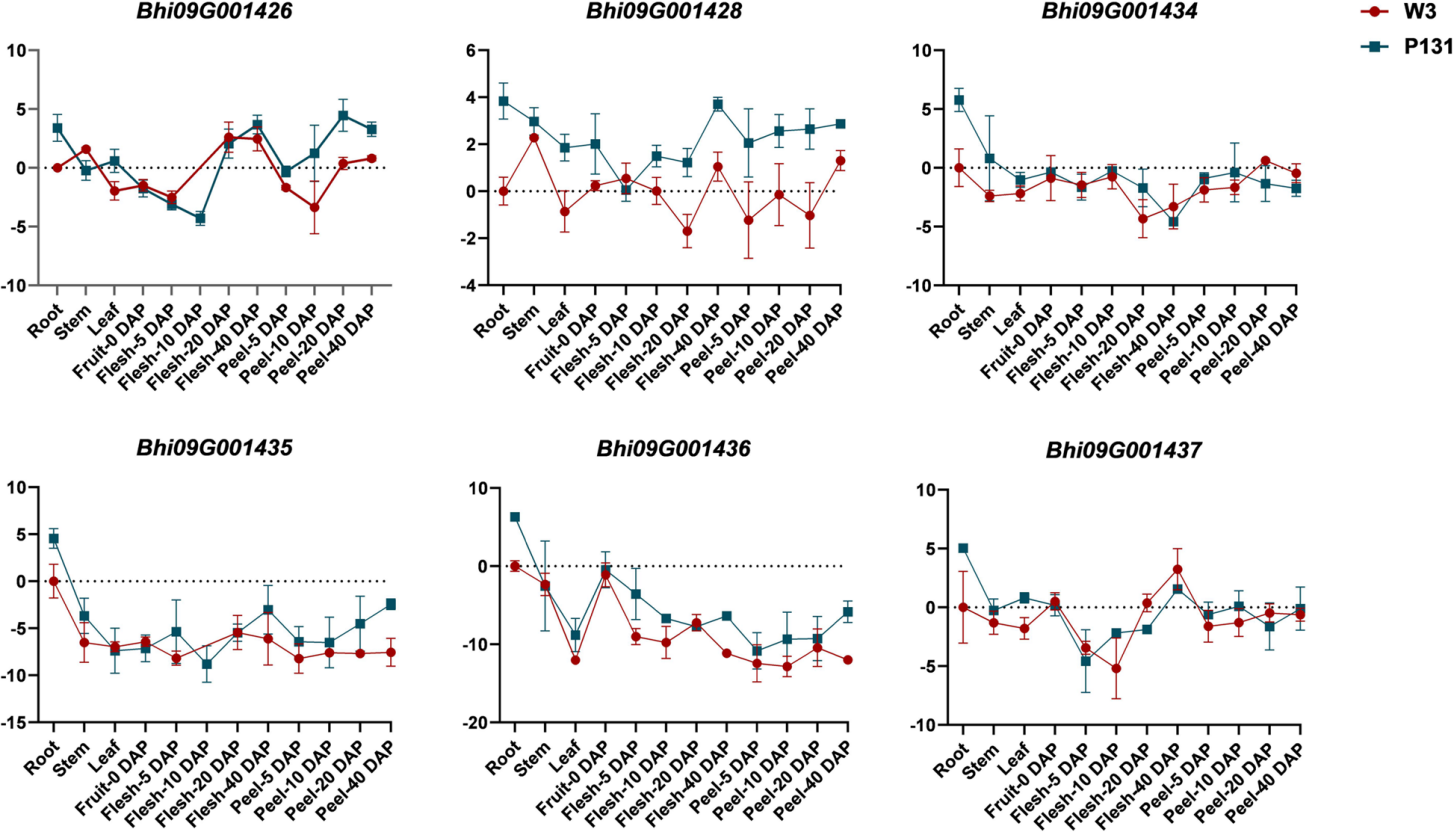
Analysis of the expression pattern of 6 genes in the *BhWAX* fine mapping interval

Based on the wax gourd reference genome [Wax gourd (B227) v1 Genome, http://cucurbitgenomics.org/v2/organism/3], totally 12 genes were identified inside the fine mapping region, including 10 with annotated function and the other 2 encoding unknown proteins (Table 3). We then analyzed the expression patterns of 6 genes (*Bhi09G001426*, *Bhi09G001428*, *Bhi09G001434*, *Bhi09G001435*, *Bhi09G001436* and *Bhi09G001437*) in different tissues and organs that are probably related to the MFCW of wax gourd using qPCR (Fig. 4). No obvious difference existed in the flesh or the peel between P_1_ and P_2_ at any time-point tested for *Bhi09G001434* and *Bhi09G001437*. Only considerable expression differences were found in the peel of fruit at 40 DAP between P_1_ and P_2_ for *Bhi09G001435*, and in the peel and the flesh of fruit between P_1_ and P_2_ for *Bhi09G001436*. Regarding *Bhi09G001426*, marked gene accumulation difference could be found between the fruit peel between P_1_ and P_2_ at 10, 20 and 40 DAP. Interestingly, the expression of *Bhi09G001428* was relatively higher in all the flesh and peel of P_1_ than in those of P_2_ at 10, 20, 40 DAP (Fig. 4). Moreover, *Bhi09G001428* also presented a gradually increased accumulation in the fruit peel and the flesh of P_1_ with the growth of the fruit. Based on the presence pattern of MFCWs in the wax gourd fruit peel, *Bhi09G001426* and *Bhi09G001428* were primarily considered as candidate genes for *BhWAX* locus.

**Table 3:** Candidate gene information in the mapping interval

Gene ID	Start (bp)	End (bp)	Description
Bhi09G001426	46,218,704	46,222,472	membrane bound O-acyl transferase
Bhi09G001427	46,225,954	46,226,163	Unknown protein
Bhi09G001428	46,261,864	46,263,661	membrane bound O-acyl transferase
Bhi09G001429	46,302,760	46,303,394	ABC1 domain-containing protein
Bhi09G001430	46,355,542	46,355,736	CCHC-type domain-containing protein
Bhi09G001431	46,359,866	46,382,186	Protein ENHANCED DOWNY MILDEW 2
Bhi09G001432	46,393,952	46,394,107	Unknown protein
Bhi09G001433	46,396,018	46,400,123	Protein ENHANCED DOWNY MILDEW 2
Bhi09G001434	46,517,195	46,521,892	Upstream activation factor subunit spp27
Bhi09G001435	46,634,565	46,635,510	AAI domain-containing protein
Bhi09G001436	46,658,408	46,659,280	AAI domain-containing protein
Bhi09G001437	46,677,870	46,685,864	Sodium/hydrogen exchanger

**Fig. 5:**
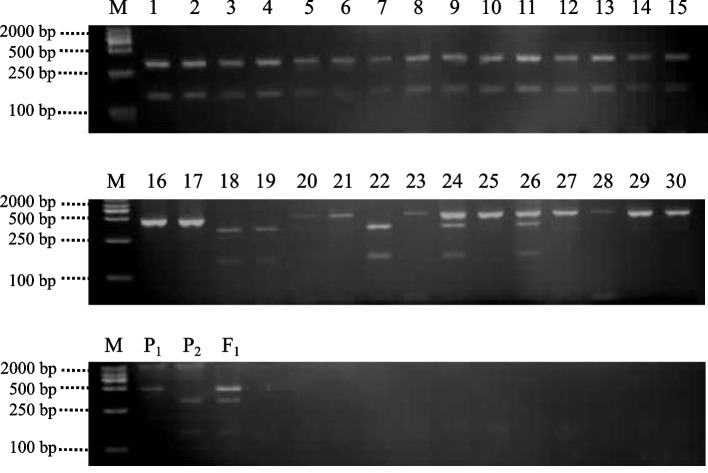
Genotype of P131, W3, F_1_ and 30 wax gourd germplasm resources shown by CAPS maker WAXS4-CAPS1. P_1_: P131, P_2_: W3. 1–30 corresponded to wax gourd germplasm resources of Table 5. The origin of the cropped gels in this figure see Additional file 2

One nonsynonymous mutation, C (P_2_) to T (P_1_) at 46,222,154 bp, was detected in gene *Bhi09G001426.* Two nonsynonymous mutation sites, C (P_2_) to T (P_1_) at 46,262,292 bp and G (P_2_) to A (P_1_) at 46,262,321 bp were detected in gene *Bhi09G001428*. Based on these three SNP sites, three cleaved amplified polymorphic sequences (CAPS) markers, WAXS1-CAPS, WAXS4-CAPS1 and WAXS4-CAPS2, were developed to test 82 F_2_ individuals, showing 70.51, 97.44 and 89.86% accuracy, respectively (Table 4). Moreover, the WAXS4-CAPS1 displaying the highest accuracy rate was further tested with 30 wax gourd germplasm resources (Table 5). All the 15 non-MFCW germplasm resources had the P_2_-like bands, while among 15 MFCW germplasm resources, 12 had the P_1_-like or heterozygous bands and the other 3 had the P_2_-like bands (Fig. 5, the original gel see Additional File 2). The accuracy of WAXS4-CAPS1 in detecting the MFCW in wax gourd germplasm resources was 80%. Altogether, we postulated that *Bhi09G001428* is the candidate gene for *BhWAX* conferring the MFCW in wax gourd.

**Table 4:** Detailed information of CAPS markers

Marker Name	SNP	Restriction Enzyme	Primer sequences (5′–3′)	Product Size (bp)			Accuracy
				P131	W3	F_1_	
WAXS1-CAPS	C-T	*Hpy*99I	F: TTTTCAGGAACTGGGTTTGG	404	239,165	404, 239, 165	70.51%
			R: CGCGCTACCTCTCTTCATCT				
WAXS4-CAPS1	C-T	*Ava*II	F: TTCACGAAATGGGCCATAGT	445	308, 137	445, 308, 137	97.44%
			R: CCACTTCGCTTCAGGACTTC				
WAXS4-CAPS2	G-A	*Bst*XI	F: TTCACGAAATGGGCCATAGT	445	271, 174	445, 271, 174	89.86%
			R: CCACTTCGCTTCAGGACTTC

**Table 5:** MFCW phenotype of 30 wax gourd germplasm resources

ID	Name	MFCW phenotype	ID	Name	MFCW phenotype
1	B96	N	16	P281	Y
2	B98	N	17	P269	Y
3	B184	N	18	PY4	Y
4	BS232	N	19	P282	Y
5	B235	N	20	P240	Y
6	B249	N	21	P109	Y
7	B274	N	22	P61	Y
8	BNH367	N	23	P257	Y
9	BS372	N	24	P264	Y
10	B430	N	25	P150	Y
11	B442	N	26	P234	Y
12	B445	N	27	P229	Y
13	B450	N	28	F3-2	Y
14	B468	N	29	P280	Y

N represents wax gourd germplasm resources without MFCW while Y represents those with MFCW

### Phylogenetic analysis of BhWAX protein

**Fig. 6:**
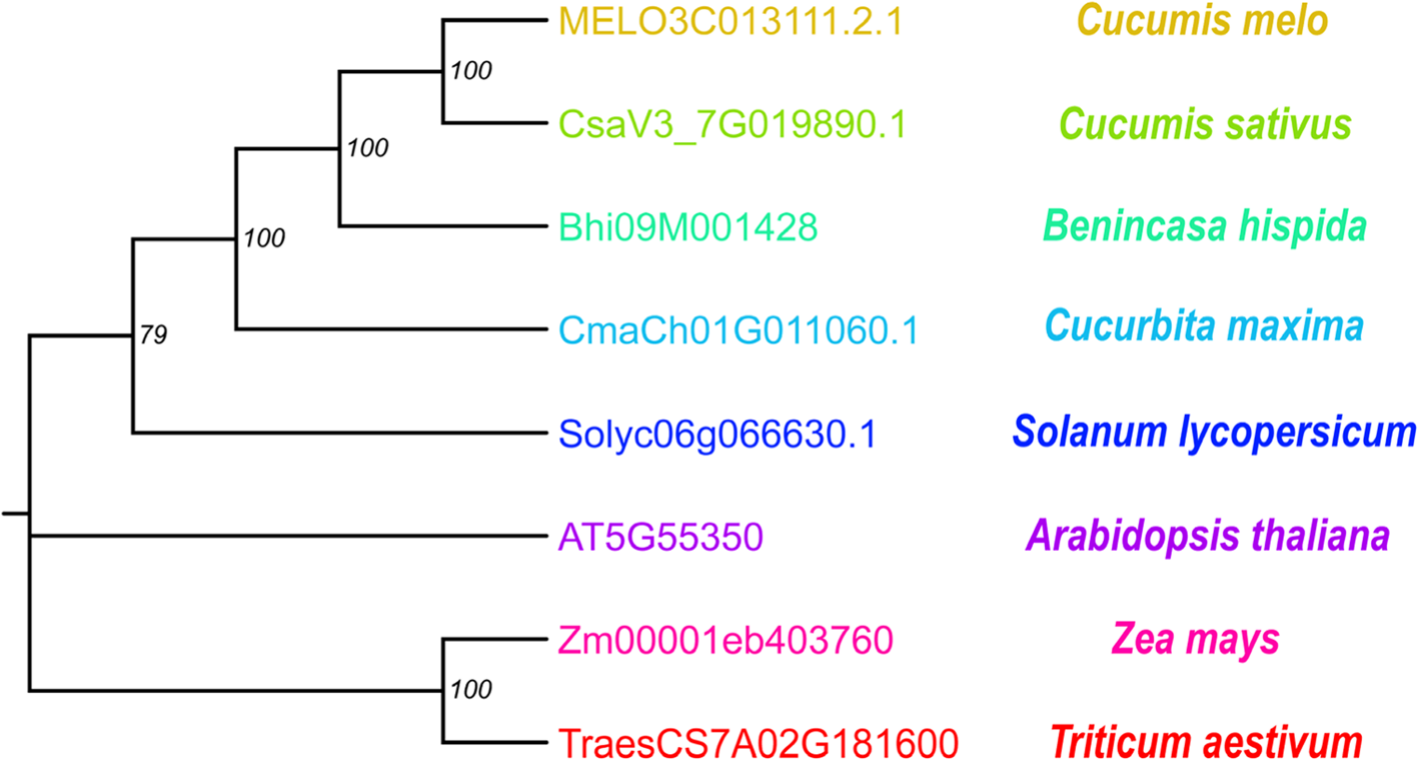
Neighbor-Joining (NJ) tree of BhWAX and its homologous proteins. The NJ tree was constructed using MEGA 11 software with default settings. Numbers at the tree forks indicated bootstrap values

*Bhi09G001428* was predicted to encode a membrane bound O-acyltransferase (MBOAT), which is homologous to AT5G55350 in *Arabidopsis*. To further dissect the relationship between *BhWAX* and homologous proteins from other plant species, a phylogenetic tree was built. The phylogenetic tree indicated that the gene product from dicotyledons formed a subclass, while those from monocotyledons formed another subclass (Fig. 6). The results also manifested that *BhWAX* exhibited a closer relation with CmaCh01G011060.1 (*Cucurbita maxima*), MELO3C013111.2.1 (*Cucumis melo*) and CsaV3_7G019890.1 (*Cucumis sativus*) (Fig. 6), indicating that this *MBOAT* gene is conserved among cucurbits.

## Discussion

Plant cuticular waxes play vital roles in protecting plants against low temperature [30], drought [31], water loss [32, 33] and many other abiotic and biotic stresses [34]. Cuticular waxes on the fruit surface could prevent plants from unlimited water loss to maintain fruit quality [35, 36] and from pathogen infection [37], and they are synthesized and change during fruit development [38]. In wax gourd, cuticular waxes are synthesized from the pedicel at around 20 DAP, whose thickness is increased with the development of the wax gourd fruit. The MFCW is an important trait that affects the commercial value of wax gourd. In China, wax gourd cultivars without MFCWs are mainly distributed in Southern China, such as Guangdong Province, Guangxi Province and Hainan Province while wax gourd cultivars with MFCW are more popular in the Southwest part, such as Yunnan Province and Sichuan Province. Furthermore, the MFCW is also a domesticated trait. MFCWs are present in wild accessions and landraces, and the cultivated non-MFCW cultivars are more likely bred from those with MFCW [29]. In this study, the GWAS demonstrated a clear signal correlated with the MFCW of wax gourd on chromosome 9. Additionally, signals on chromosome 2 and 6 could also be seen, indicating that the MFCW of wax gourd could be controlled by deferent genes dependent on germplasm resources.

In the present study, the MFCW of wax gourd could be controlled by a single dominant gene *BhWAX*, which is in accordance with most of previous studies on other plant species [19, 23, 39]. Recently, BSA-seq has been widely used in the rapid and efficient identification of QTL or genes related to specific traits [40–42]. The combination of BSA-seq with fine mapping using 2,487 F_2_ individuals, the candidate gene for *BhWAX* was mapped to a 0.51 Mb interval. During the process of fine mapping, no recombination was found between marker SNP1 and SNP15, which could be attributed to the short distance between this region and the centromere of the chromosome. Because of the large genome size of wax gourd (913 Mb) [29], only 12 genes were found in the interval. Among these genes, *Bhi09G001426* and *Bhi09G001428* had relatively higher expression levels in the fruit peel of wax gourd inbred line with MFCW than in the line without MFCW at 20 and 40 DAP. This expression pattern is in consistent with the timing of wax formation on the wax gourd fruit peel. Therefore, we primarily considered these two genes as candidates. Further, CAPS markers were developed based on the nonsynonymous mutation sites inside two genes, and *Bhi09G001428* was finally proposed as the candidate gene because of its high efficiency in determination of MFCW trait in both wax gourd F2 population and germplasm resources.

*Bhi09G001428* encodes a MBOAT gene, also known as Wax Synthase (WS) [43]. In a previous study, a total of 136 MBOAT genes were identified from 15 plant species [44]. MBOAT genes contain MBOAT domains [45] and were firstly identified in *Drosophila* [46]. However, the function of MBOAT genes remain largely uncharacterized as there are only few relevant reports. The heterelogous overexpression of a WS gene from Jojoba greatly increases the wax content in the seed oil [47]. The heterelogous overexpression of a sunflower WS gene in Saccharomyces cerevisiae can accumulate C-32 to C-36 wax esters by feeding C16 and C18 fatty alcohols combined with 16:0, 18:0 fatty acyl CoAs [48]. In microalga, a WS gene exhibits wax synthase activity and diacylglycerol acyltransferase activity, thus promoting triacylglycerol accumulation [43]. Altogether, the above studies indicate that MBOAT genes function in the synthesis of wax ester components, which are also main compounds of plant cuticular waxes. Therefore, it is reasonable for us to propose that *Bhi09G001428* act as the candidate conferring the MFCW in wax gourd.

MFCW trait differs among wax gourd germplasm resources, some with thin powder while many other with thick powder, and we speculate that *Bhi09G001428* is not the only gene contribute to MFCW trait in wax gourd. Except for the gene on chromosome 9, other loci, for instance on chromosome 6 and chromosome 2, were detected by GWAS (Fig. 2). Therefore, further work are still needed to be done to explore other genes related to MFCW in wax gourd.

## Conclusion

In this study, we found that the MFCW of wax gourd was dominantly controlled by a single gene *BhWAX*. Genetic mapping delimited *BhWAX* to a 0.51 Mb interval on chromosome 9 containing 12 genes. Based on the gene annotations and expression patterns, a MBOAT gene was proposed as the candidate for *BhWAX*. Further, an efficient CAPS marker was developed to determine the MFCW of wax gourd. This study is the first report about the identification of the gene controlling the *MFCW* and development of the trait-related molecular markers in wax gourd, which will not only contribute to the functional validation of the gene and marker-assisted breeding of wax gourd, but also lay a foundation for analysis of its evolutionary significance among cucurbits.

## Methods

### Plant materials

Two wax gourd inbred lines P131 (P_1_, with MFCW) and W3 (P_2_, without MFCW) were selected as experimental materials and crossed to produce F_1_ hybrid. In the spring of 2019, F_1_ underwent self-pollination to produce F_2_, and backcrossed with P_1_ and P_2_ to produce BC_1_P_1_ and BC_1_P_2_, respectively. The P_1_, P_2_, F_1_, BC_1_P_1_, BC_1_P_2_ and 574 F_2_ individuals were planted in the autumn of 2019. Besides, 2,487 F_2_ individuals were firstly planted in 96-well trays and the recombinants were later transplanted to the field. All plant materials were grown in the experimental field of Vegetable Research Institute, Guangdong Academy of Agricultural Sciences.

### Phenotypic data collection

The MFCW of each tested individual was observed visually and scored by three people at the fruit maturity stage [around 50 days post pollination (50 DAP)]. The fruit with cuticular waxes was scored as "1" point while that without was scored as "0" points.

### Deoxyribonucleic acid (DNA) and Ribonucleic acid (RNA) extraction

After collection, young leaf samples of P_1_, P_2_, F_1_ and all F_2_ individuals were frozen in liquid nitrogen and kept in a -80ºC freezer for further use. The DNA was extracted as described in literature [49]. At different developmental stages (0, 5, 10, 20 and 40 DAP), the fruit peel and flesh as well as root, leaf and stem were collected from P_1_ and P_2_ for RNA extraction, each with three biological replicates. Subsequently, the RNA was extracted using TransZol Up Plus RNA Kit (TransGen, Beijing, China) according to manufacturer's instructions.

### GWAS

The existence of MFCWs and re-sequencing data of 146 wax gourd germplasm resources were collected from our previous study [29] for GWAS analysis. A mixed linear model (MLM) was applied for relevant signal detection. *p* value indicating the correlation between each single nucleotide polymorphism (SNP) and the MFCW was calculated with TASSEL v5.0 [50]. Finally, the Manhattan plots were graphed using CMplot [51].

### Bulked segregant analysis (BSA) combined with sequencing (BSA-seq) analysis

For the bulked segregant analysis (BSA), two bulks, F_2_-1 and F_2_-0, were constructed by pooling equal quantities of genomic DNAs from 30 F_2_ plants with cuticular wax and 30 F_2_ plants without cuticular waxes, respectively. The high-throughput genome sequencing data of two bulks and two parental lines based on pair-end libraries were sequenced using HiSeq X10 (Illumina Inc., San Diego, CA, USA) and NGS platforms (Genedenovo, Guangzhou, China).

### Genetic mapping of BhWAX locus

According to BSA-seq analysis, 159 InDel sites from chromosome 9 were selected for developing InDel markers. The InDel markers were then validated using P_1_, P_2_ and F_1_, and polymorphic markers were used to genotype 145 F_2_ individuals (Detailed marker information is shown in Additional File 1). Next, linkage map was prepared using JoinMap4.0, and gene mapping was conducted using QTL IciMapping Version 4.2 [52]. Later, 2,487 F_2_ individuals were genotyped by two flanking markers C9-46.175 and C9-47.164. Inside the primary gene mapping interval, 24 SNP sites were further selected for recombination screening. Briefly, primers were designed to amplify fragment containing the SNP sites, and the amplicons were sent for sequencing to obtain the SNP information of the recombinants (Detailed marker information is shown in Additional File 1). Based on the genotype and MFCW trait of the recombinants, the final candidate region of *BhWAX* was confirmed.

### Quantitative real-time polymerase chain reaction (qRT-PCR) analysis

QRT-PCR analysis was performed using TB Green™ Premix Ex Taq™ II (Tli RNaseH Plus, Takara, Kyoto, Japan) kit according to manufacturer's instructions. The reaction was performed in a CFX96 Real-Time PCR Detection System (Bio-Rad) containing 50 ng of cDNAs, 100 nM of each primer in a reaction system (10 μL). Three biological replicates and three technological replicates were set for each sample. With the wax gourd *UBQ* gene as internal control, the expression of genes was calculated using 2^−ΔΔCt^. Primers used in this analysis are listed in Additional File 1.

## Supplementary Information

**Additional file 1.** The detailed primer information of markers used in initial genetic mapping and fine mapping of BhWAX, and primers of qRT-PCR used in the study. 

**Additional file 2**. Original gel of the cropped gels in Fig. 5.

## Data Availability

The re-sequencing data and MFCW trait of wax gourd germplasm resources used for GWAS is available as described (Xie et al. 2019). Other materials generated and analyzed in the study are available from the corresponding author on reasonable request.

## Abbreviations

BSA: Bulked segregant analysis
CAPS: Cleaved Amplified Polymorphic Sequences
DAP: Days after pollination
GWAS: Genome wide association study
MBOAT: Membrane bound O-acyl transferases
MFCW: Mature fruit cuticle wax
