# Supplementary figures and images for: Genome-wide association study and genetic mapping of BhWAX conferring mature fruit cuticular wax in wax gourd
Source: BMC Plant Biol. 2022 Nov 19;22:539. doi: 10.1186/s12870-022-03931-z (PMC9675113; doi:10.1186/s12870-022-03931-z)

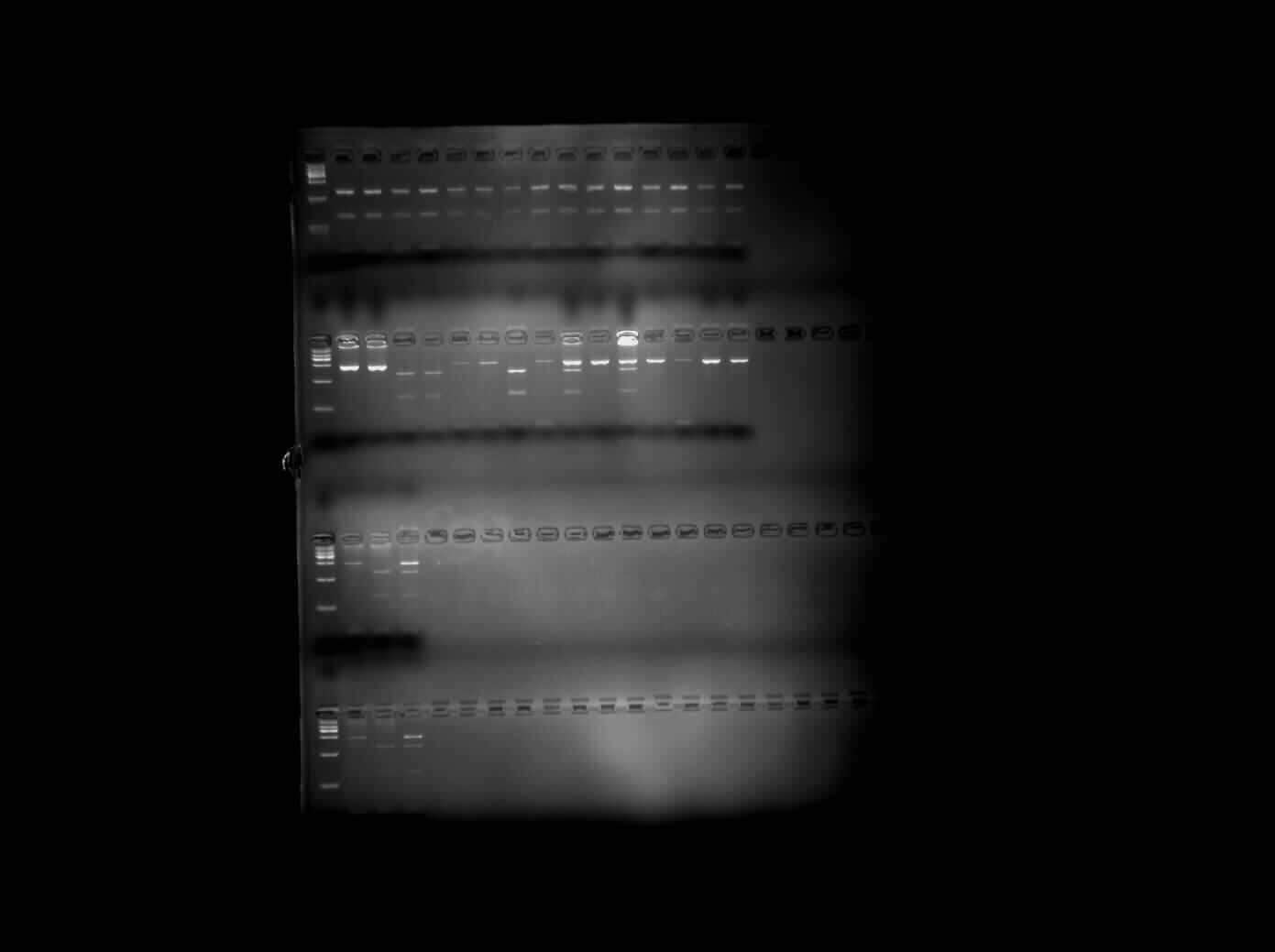


**Additional file 2** Original gel of the cropped gels in Fig. 5.

Supplement: Supplementary file 2 — Additional file 2. Original gel of the cropped gels in Fig. 5. [file 12870_2022_3931_MOESM2_ESM.doc]
